# Bioavailability of generic 0.05% difluprednate emulsion in the aqueous humor, cornea, and conjunctiva of New Zealand rabbits after a single dose compared with commercial difluprednate

**DOI:** 10.1186/s12348-017-0127-2

**Published:** 2017-03-21

**Authors:** Arieh Mercado-Sesma, Angélica Contreras-Rubio, Leopoldo Baiza-Durán, Oscar Olvera-Montaño, Mónica Miranda-Robles, José Bonilla-García

**Affiliations:** 1 Preclinical Research Department, Laboratorios Sophia S.A de C.V., Zapopan, Jalisco México; 2 Departament of Health-Disease Sciences as Individual Process, Centro Universitario de Tonala. Universidad de Guadalajara, Tonalá, Jalisco México

**Keywords:** Difluprednate, Pharmacokinetic, Difluprednate bioavailability, Ophthalmic corticosteroids

## Abstract

**Background:**

To determine the concentration after a single dose of generic 0.05% difluprednate and commercial difluprednate in the aqueous humor, cornea, and conjunctiva of New Zealand rabbits, a preclinical study in 72 male New Zealand white rabbits was performed. A single dose (50 μL) of two 0.05% difluprednate ophthalmic formulations was instilled in both eyes. Conjunctiva, cornea, and aqueous humor samples were collected at nine time points over 8 h (four animals per time point). The active metabolite of difluprednate, 17-difluoroprednisolone-butyrate (DFB), concentrations was quantified using HPLC.

**Results:**

Measurable levels of DFB were quantified in all three ocular tissues. After a single instillation, the highest concentration of difluprednate was found between 30 and 60 min in the conjunctiva, cornea, and aqueous humor, respectively. There was no significant difference between both formulations in any tissue at any time point. After 3 h, no metabolites of either emulsion were found in any tissue.

**Conclusions:**

Difluprednate penetrates into different ocular tissues. Generic difluprednate has a similar pharmacokinetic profile compared with commercial difluprednate.

## Background

Despite of the eye having immune privilege [1, 2] exist a lot of situations where an inflammatory response can be ignited. If an uncontrolled inflammatory ocular response appears, it can lead a patient to present unpleasant symptoms and signs: pain, edema, discomfort, photophobia, hyperemia, synechiae, and others. But above all can be associated with some complications, corneal edema, posterior capsule opacification, or cystoid macular edema [3–5]. Fortunately, it is possible to limit this obnoxious experience by the employment of anti-inflammatory medications, and their prophylactic use is a standard practice. Among anti-inflammatory medications, corticosteroids are the cornerstone in the treatment of this pathophysiologic process. However, most corticosteroids have a low ocular bioavailability, due in part to the characteristics of the cornea, limiting their effect [6, 7].

Difluprednate is a prednisolone derivative with structural modifications (fluorination at the C6 and C9 positions and replace 17-hydroxyl group with butyric acid) that enhanced and augmented its potency and anti-inflammatory activity, respectively. Furthermore, the substitution of the 21-hydroxyl group with acetic acid increased the corneal affinity and absorption [8, 9].

Despite to be approved in other countries since 2008, in Mexico and Latin America, difluprednate ophthalmic emulsion is not commercially available. Hence be important to develop a new therapeutic option for the treatment of inflammatory diseases.

The objective of the present study was to compare the ocular pharmacokinetics of generic difluprednate and its commercial presentation in an animal model.

## Methods

### Study design

The study was a double-blinded preclinical study. Both the ophthalmologist investigator and biostatistician were blinded to the study medication.

### Animal model

Seventy-two male New Zealand white rabbits, weighing between 2–3 kg, obtained from a local farm were used in this study. Animals with ocular abnormalities were excluded from the study. All rabbits were housed individually under controlled conditions, which included temperature ranging between 16 and 26 °C, humidity between 40 and 70%, and cycles of light/dark of 12/12 h. Water ad libitum was arranged. Food consisted of standard alfalfa pellets. They were randomly allocated to both groups, so as to get four animals per time point.

### Drug administration and sample collection

Animals were randomized to two groups to receive difluprednate emulsion 0.05% manufactured by two different laboratories: PRO-145 (Laboratorios Sophia S.A. de C.V, Guadalajara, Jalisco, México) or Durezol® (Alcon Laboratories, Inc., Forth Worth, Texas, USA). A single 50-μL eye drop of each drug formulation was instilled into the superior bulbar conjunctiva of both eyes. Sampling for drug concentrations were performed at the following time points after drug administration: 15, 30, and 60 min; 1.5, 2, 3, 4, 6, and 8 h (*n* = 4 rabbits per time point). The tissues taken for pharmacokinetic analysis were bulbar conjunctiva, cornea, and aqueous humor. The samples were obtained after animals were euthanized by an overdose of pentobarbital (100 mg/kg). Immediately after death, the bulbar conjunctiva, cornea, and aqueous humor were dissected, stored (−20 °C), and processed separately.

### Drug assay

The samples were assayed using a validated high performance liquid chromatography method (HPLC; Waters corporation, Milford, MA, USA) The analytical column was C18 Gemini (Phenomenex NS655712-5) with a 5-μm particle size and 150 × 4.6 mm internal diameter, using a mobile phase of phosphate buffer and acetonitrile. The wavelength of detection was 240 nm. The lower limit of detection was 4 ng/mL in the aqueous humor and 23.7 ng/mL in the cornea/conjunctiva.

### Statistical analysis

The concentration values are showed in mean and SD. Pharmacokinetic parameters (C_max_, T_max_, and T_1/2_) were calculated by non-compartmental analysis using WinNonlin version 2.1 (Pharsight Corporation, Mountain View, CA, USA). The difference between groups was analyzed by Mann-Whitney test using SPSS version 19 (IBM Corporation., Armonk, NY, USA). A value *p* < 0.05 was considered to be statistically significant.

## Results

**Fig. 1:**
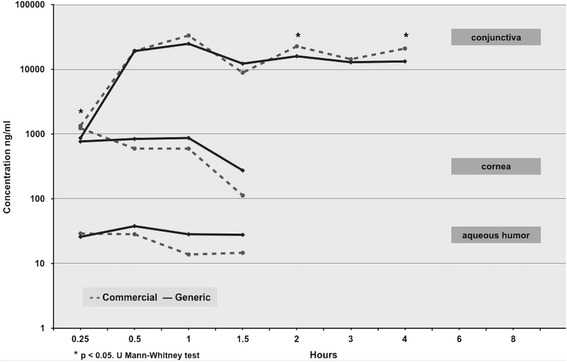
Bioavailability of 0.05% generic vs commercial difluprednate in different ocular tissues

Concentrations of 17-difluoroprednisolone-butyrate (DFB) were detected in all tissues. The highest concentration was observed in the conjunctiva at 0.5–1 h in both formulations, and the lowest was detected after 4 h. As expected, the concentration of DFB was different in the aqueous humor, cornea, and conjunctiva. There were no differences between the determinations of both formulations in the cornea and aqueous humor at any time point. There were statistical differences in DFB concentration measured in the conjunctiva at times 15 min and 2 and 4 h; however, after 6 h, both formulations were undetectable Fig. 1.

The pharmacokinetic parameters were similar in both formulations. The T_max_ was found at 0.5–1 h in all tissues. There was no significant difference between formulations in terms for any parameter Table 1.

**Table 1:** Pharmacokinetic parameters of difluprednate in the aqueous humor, cornea and conjunctiva after a single instillation

PK parameter	Aqueous humor		Cornea		Conjunctiva	
	Generic	Commercial	Generic	Commercial	Generic	Commercial
T_max_	30	30	15	15	60	60
C_max_	35	27	1072	1255	25,288	31,351
T_1/2_	2.1	2.1	1.1	1.4	4.6	4.2

Concentration in nanograms per milliliter

*T*_*max*_ time of maximum concentration, *C*_*max*_ maximum concentration, *T*_*1*/*2*_ half-life

## Discussion

Corticosteroids are a widely used topical ocular therapy. They are an important component of the treatment of diseases in which inflammatory response plays a central role, suppressing cellular infiltration and increasing the synthesis of lipocortins, mainly [6]. Difluprednate, a corticosteroid, mediates its anti-inflammatory effects through the glucocorticoid receptor after intracellular distribution [10]. However, several factors are involved in ophthalmic drug efficacy, diffusion and distribution to targeted ocular tissues are of the most important [11, 12]. Due to its chemical structure, difluprednate ophthalmic emulsion has a high lipophilicity, which facilitates passage through the membranes, allowing a significant distribution to the anterior and posterior segments, unlike early generations of corticosteroids [13], and clinical efficacy in medical settings [14, 15].

Ocular pharmacokinetics after a single instillation of difluprednate has been studied in rabbits. T_max_ in both studies was similar in aqueous humor, in spite of having used different methods of quantification [13, 16]. In the present data, T_max_ was similar in aqueous humor according to mentioned below.

The half-life (T_1/2_) reported in the aqueous humor, conjunctiva, and cornea [13] was similar to the present results.

Notwithstanding that C_max_ determined in the aqueous humor and cornea was similar that described in the previously mentioned, this parameter differed in conjunctiva’s quantification. In the present study, the values were higher than those reported previously.

These discrepancies could be due to the methods used. Tajika et al. [13] quantified DFB concentration by HPLC using a radio labeled with tritium in contrast with the present data.

However, in the present study, the results were not dissimilar according to the comparator. Therefore, distribution of both formulations had the equivalent pharmacokinetic profile. Although the cornea is a major barrier, both formulations showed similar concentrations in all tissues at the present study. There are no other reports with similar methods evaluating the concentration of difluprednate in the conjunctiva.

The present data confirm that pigment does not affect the bioavailability of difluprednate. Difluprednate is distributed to the anterior and posterior segments via both the transcorneal and non-corneal absorption route [13, 16]. However, the distribution to anterior-posterior retina/choroid was not evaluated, and this is a weakness of the present study.

Although there was no significant difference in the pharmacokinetic profile between formulations used, the pharmacodynamic characteristics of generic difluprednate have not been determined yet. More studies are required to determine the security and efficacy of difluprednate in Mexican population.

## Conclusions

Generic difluprednate has a similar pharmacokinetic profile compared with commercial difluprednate. Pigment does not affect the bioavailability of difluprednate which is distributed to the anterior and posterior segments via both the transcorneal and non-corneal absorption route.

## Abbreviations

ARVO: Association for Research in Vision and Ophthalmology
CICUALLS: Internal Committee for the Care and Use of Laboratory Animals of Sophia Laboratories
C_max_: Maximum concentration
DFB: 17-Difluoroprednisolone-butyrate
GLP: Good Laboratory Practice
HPLC: High performance liquid chromatography
T_1/2_: Half-life
T_max_: Time of maximum concentration
